# The Drop-in-Drop Encapsulation in Chitosan and Sodium Alginate as a Method of Prolonging the Quality of Linseed Oil

**DOI:** 10.3390/polym10121355

**Published:** 2018-12-06

**Authors:** Szymon Mania, Robert Tylingo, Anna Michałowska

**Affiliations:** 1 Chemical Faculty, Department of Chemistry, Technology and Biotechnology of Food, Gdansk University of Technology, 80-233 Gdansk, Poland; robertt@pg.edu.pl; 2 UCB Pharma, 216 Bath Rd, Slough SL1 3WE UK; anna.s.michalowska@gmail.com

**Keywords:** chitosan, coaxial technique, core-shell encapsulation, linseed oil, sodium alginate

## Abstract

Nowadays, the encapsulation of sensitive products by various techniques has become popular as a promising preservation method. In particular, this applies to oils with a high content of unsaturated fatty acids and a high susceptibility to deterioration. This work presents the possibility of using a chitosan and sodium alginate in the form of a hydrogel membrane to protect food ingredients such as linseed oil, which is stored in an aquatic environment. The obtained results showed the high efficiency of the coaxial method encapsulation, which did not affect the quality of the oil measured after encapsulation. The greatest protective effect was observed in the linseed oil–chitosan membrane system, in which the primary and secondary oxidation products content were 88% and 32% lower than in the control sample, respectively. The smallest changes of the fatty acid profile, conjugated dienes, and trienes were observed in the chitosan capsules with linseed oil compared to the control sample.

## 1. Introduction

Research on consumer attitudes and behaviors regarding the food market has shown a growing interest in the quality and health safety of food products and their stability throughout the shelf life. Consumers often can not assess the deterioration of product quality. Therefore, protecting the product and maintaining the proper activity of functional ingredients in this period belongs to the producers [1,2]. One example of such ingredients is polyunsaturated fatty acids (PUFAs), especially n-3 fatty acids, whose average intake in humans is below the recommended level. Many clinical and epidemiologic studies have shown the positive effect of n-3 fatty acids on infant development; cancer; cardiovascular diseases; and more recently, in various mental illnesses, including depression, attention deficit hyperactivity disorder, and dementia [3,4,5]. One rich source of n-3 acids is linseed oil, which may contain up to 90% PUFAs, of which about 50% is α-linolenic acid [6]. However, the high content of PUFAs containing double bonds determines their low stability, which is a significant limitation in the commercialization of linseed oil on a large scale. Oxidative transformations of PUFA initiated by the atmospheric oxygen or light radiation cause not only adverse sensory changes in the oil, they also lower its nutritional value. Moreover, the formation of secondary oxidation products (SOPs) that are harmful for the human health can occur such as: monometric decomposition products (epoxides, hydroxyl, and cyclic derivatives of lipid hydroperoxides), low molecular weight compounds (aldehydes, ketones, alcohols, and hydrocarbons) and high molecular complex products (aliphatic, monocyclic, bicyclic, dimeric, and trimeric reaction products polymerization or copolymerization) [7].

One of the potential solutions to delay the oxidative changes in highly unsaturated oils is the encapsulation process. Still, the most commonly used industry technique for oil encapsulation is emulsion spray drying [8]. However, Nazzaro et al. [9] confirmed that the main factor associated with the oxidation of oil encapsulated by this technique is the increased availability of oxygen during the formation of oil emulsion with the carrier. This is followed by high temperature in the drying chamber (about 200 °C), when the carrier solvent is evaporated from the ready emulsion. In recent years, coaxial encapsulation has been particularly appreciated. The transport of the carrier and encapsulated compound along the same axis allows forming the drop-in-drop structures without an emulsification step and the use of high temperatures to get a dry form of capsules, which was also confirmed in our earlier work [10].

In the scientific literature, there are works on attempts to use coaxial encapsulation in prolonging the freshness of food ingredients, including edible oils. However, in most cases, these works concern dry capsules on a microscale size [8,11,12,13,14].

Very popular carriers in encapsulation processes are natural polymers, especially proteins and polysaccharides including agarose, alginate, carrageenan, chitosan, gellan gum, hyaluronic acid, collagen, elastin, gelatin, fibrin, and silk fibroin [15]. Their application is associated with several advantages such as biocompatibility, biodegradability, the ability to receive from renewable raw materials, and good susceptibility to physical and chemical modifications, which creates great opportunities in various fields of science and industry. Biopolymer systems are used in biomedical and biotechnological applications, such as xenotransplantation, the maintenance of stem cell phenotype, anti-cancer therapies, and the bioprinting of three-dimensional scaffolds for tissue engineering and regenerative medicine [15,16,17]. Encapsulation has also become a convenient option in the food industry; a wide variety of foods is encapsulated, including flavoring agents, acids, bases, artificial sweeteners, colorants, preservatives, leavening agents, antioxidants, agents with undesirable flavors, odors, and nutrients [18].

The aim of our work is to use chitosan and sodium alginate in the hydrogel membrane form, which can protect food ingredients, as in the example of linseed oil stored in the aquatic environment. According to the research hypothesis, the chitosan hydrogel as a capsule shell is able to create the barrier for linseed oil core as a rich source of polyunsaturated fatty acids. So far, the protective effect of chitosan and alginate in the hydrogel form has not been tested in such a matrix.

Our work shows the influence of linseed oil encapsulation on its quality immediately and after the oil encapsulation in chitosan or alginate membrane, and the effect of oil storage in encapsulated form in the aqueous environment. As a control to the tests carried out, capsules with refined rapeseed oil were used, in which the content of n-3 acids is about five times lower than in linseed oil [19]. The obtained results indicate the potential of this new method, which can be an alternative technique for the encapsulation of PUFA-rich oils as a functional addition to food with a high water content.

## 2. Materials and Methods

### 2.1. Materials

Medium molecular weight chitosan polymer (MMW) with 75–85% deacetylation degree (viscosity 200 ÷ 800 cps 1% concentration solution in 1% acetic acid at 25 °C) and sodium alginate (viscosity 5.0 ÷ 40.0 cps and pH = 5 ÷ 8 for 1% concentration solution in water at 25 °C) were obtained from Sigma Aldrich (Saint Louis, MO, USA). Aqueous lactate acid (CHEMPUR, Piekary Śląskie, Poland) solution was used as a solvent for the chitosan polymer. Capsule core and the reference samples were the cold pressed linseed oil “Wielkopolski” produced by SemCo^®^ (Śmiłowo, Poland) and refined rapeseed oil “Kujawski” produced by Z.T.Kruszwica (Kruszwica, Poland). For hardening the capsules, ethanol (96% *v*/*v*, Avantor Performance Materials Poland S.A., Gliwice, Poland), sodium hydroxide, and calcium lactate (P.P.H. Stanlab, Lublin, Poland) were used. For the encapsulation process, the compressed nitrogen (N_2_ 4.0) was used. For the other chemical determinations, we used: methanol, chloroform, acetic acid (Avantor Performance Materials Poland S.A., Gliwice, Poland), sodium thiosulfate, potassium iodide (Sigma Aldrich, Saint Louis, MO, USA), starch (P.P.H. Stanlab, Lublin, Poland), hexane, n-heptane, and tetrahydrofuran for chromatography Merck KGaA Darmstadt, Germany). Carbon dioxide was bought from “Linde” (Gdansk, Poland). The 3-(4,5-dimethylthiazol-2-yl)-2,5-diphenyltetrazolium bromide (MTT), medium, antibiotics, and supplements that were necessary for cell culture were obtained from Sigma-Aldrich (St. Louis, MO, USA). L929 cells (adult mouse fibroblast cell line) were obtained from ATCC (American Type Culture Collection). All of the chemicals were of analytical grade, and no further purification was required.

### 2.2. Methods of Shell and Preparation of Hardening Solutions

As a shell of the capsules, the 2% chitosan in 0.1-M lactic acid solution or 2% sodium alginate in distilled water was used. In this case, an appropriate weight of the polymer was successively added to the lactic acid solution or distilled water respectively, during continuous mixing (300 rpm, Heidolph RZR 2052 control) to obtain the clear solution. The solution of each polymer was ready to use after two hours of incubation at room temperature (the time required for degassing). Chitosan and alginate capsules were collected using 50% (*v*/*v*) ethanol solution containing 5% sodium hydroxide (having regard to the phenomenon of volume contraction) and 3% calcium lactate solution, respectively. The ethanol solution was obtained by gradually dissolving sodium hydroxide in ethanol and diluting the solution with an amount of distilled water in a vessel placed in an ice bath. The calcium lactate solution was obtained by dissolution of the appropriate weight of salt in distilled water.

### 2.3. Preparation of Chitosan/Alginate Microcapsules with Oil

For the production of capsules, we used the coaxial technique with modification of the manner of transport solutions, preventing their mixing, according to Tylingo et al. (Video S1) [10]. To produce the drop-in-drop capsules, the oil and shell solution was transferred into conical flasks. High-pressure nitrogen was used to drive the oil and shell solution into and through the nozzle. The flow rate of them was controlled by two precision rotameters with valves (BROOKS INSTRUMENTS SHO-RATE^TM^ 250 AD6021NMANB, Hatfield, PA, USA). The launch of the equipment consisted of unscrewing the valves of compressed nitrogen bottles and stabilizing the solution flow-out so that a double-layer structure of drops was obtained. A glass plate on a magnetic stirrer (15-cm diameter) filled with 250 mL of hardening solution was kept just below the tip of the compound nozzle. The distance from the tip to the collecting plate was fixed at five centimeters during the experiment. The fall drop to the collection plate was caused gravitationally. Formed capsules were collected in hardening solution and stirred for two hours for full cure. Then, the chitosan capsules were thoroughly rinsed with distilled water to reach pH 7. The alginate capsules were also rinsed with distilled water at the same time as the chitosan capsules to remove the residue of lactate. Until further measurement, capsules were stored in distilled water at 4 °C.

### 2.4. Determination of Encapsulation Efficiency and Capsules Size Distribution

The encapsulation efficiency (EE%) is defined as the value of the incorporated oil detected in the prepared capsules over the initial value that was used to make the capsules. The encapsulation efficiency was calculated by the following formula:

EE (%) = (OC/Md) × 100,(1)

where OC is the amount of the oil determined in 100 g of hydrogel capsules based on Equation (2), and Md is the total quantity of the oil used during the preparation of 100 g of hydrogel capsules measured gravimetrically during encapsulation.

The size distribution of moist oil–chitosan and oil alginate capsules was observed by an optical microscope (DELTA optical EVOLUTION 100 (Warszawa, Poland)) with an HDCE-50B Digital Camera. Before the measurements, each capsule was filtered from the excess water on the filter paper, and then placed on a microscope glass slide. For monitoring the diameter of the capsules, ScopeImage Plus software with the above-mentioned equipment was used. The final diameter is the average value of 100 readings performed on the same sample.

### 2.5. Determination of Oil Content inside the Capsules

Oil content in capsules was determined using extraction with hexane. Firstly, the small amount of hydrogel capsules was weighed and freeze-dried (CHRIST Alpha 2–4 LSC, Osterode am Harz, Germany). Dry capsules were destroyed and quantitatively transferred to cellulose thimbles. Extraction was carried out in a glass set that consisted of a 250-mL round-bottom flask and a reflux condenser. The round-bottom flask was weighed, and in the next step, it was filled with 50 mL of hexane. Cellulose thimble was placed in the same flask above the liquid surface. After reaching the boiling temperature of the hexane, extraction was conducted for 90 minutes. After cooling the glass set, the extraction solvent was evaporated from the round flask using a rotary vacuum evaporator (BÜCHI Rotavapor R-134 with BÜCHI Waterbath B-480, Flawil, Switzerland). In order to remove the residual organic solvent, the inside of the flask was dried further by using the nitrogen stream. The flask with oil was then weighed. The oil content was calculated, and the weight of the hydrogel capsules was determined by the following formula:

OC (%) = ((M2 − M1)/Mc) × 100,(2)

where M2 is the mass of oil with the round-bottomed flask after its extraction and solvent evaporation in grams, M1 is the mass of the empty round-bottomed flask in grams, and Mc is the mass of capsules before drying used in the dry weight determination in grams. The oil-to-polymer ratio was calculated according to the method proposed by Tylingo et al. [10].

### 2.6. Assessment of the Encapsulation Impact on the Oxidative Stability of the Oil

#### 2.6.1. Determinations of Oil Quality

The quality of refined rapeseed oil and cold-pressed linseed oil in the free and encapsulated form was tested based on the content of primary oxidation products, including: hydroperoxides, which determined the peroxide value (PV) [20]; secondary oxidation products, which determined the anisidine value (AV) [21]; the content of free fatty acids, which determined the acid value (AcV) [22], and the fatty acid profile determined by the Gas Chromatography method (Perkin Elmer, Waltham, MA, USA) according to the following methodology.

The method of transesterification by methyl alcohol in the presence of the alkaline catalyst at low temperature [23] was used to convert fatty acids to methyl esters (FAMEs). The FAMEs were analyzed with a Perkin Elmer Autosystem XL gas chromatograph, equipped with a 30-m DB-23 silica capillary column (Agilent J&W Scientific, Santa Clara, CA, USA) of 0.25-mm internal diameter and a film-shelling thickness of 0.25 mm. The helium carrier-gas column flow rate was 0.91 mL/min. A split–splitless (60:1) injector at 250 °C and flame ionization detector (FID) at 250 °C were used. The column temperature, after an initial isothermal period of five minutes at 120 °C, was increased to 180 °C at a rate of 1.5 °C/min. and maintained for 25 minutes. The temperature was increased again to 210 °C and was maintained for 30 minutes. Other quality attributes such as conjugated dienes and trienes were expressed by the absorption coefficient $E_{1\;\mathrm{cm}}^{1\%}$ at λmax 232 nm and 268 nm using UV–VIS Genesis 2 (Milton Roy, Houston, TX, USA) spectrophotometer [24]. The tocopherols were measured according to the PN-EN ISO 9936:2016 [25] standard by using the HPLC Agilent 1200 Series system with a photodiode array detector: E_x_ = 295 nm, E_m_ = 340 nm. The analysis of the samples was carried out in the normal phase with a LiChro CART 125 × 4 DIOL (five-μm) column. As mobile phase, 3.85% (*v*/*v*). tetrahydrofuran in n-heptane was used, the volume of injection was five μL, and the flow rate was 0.8 mL/min. The analysis time and column temperatures were 20 minutes and 30 °C respectively. 

For the determination of the quality of encapsulated oil, in the methodology, an additional stage of the oil isolation from the capsule interior was applied. In the case of PV, AV, AcV, dienes, trienes, and tocopherols, the appropriate weight of capsules in the mortar was destroyed. The released oil was quantitatively transferred to the laboratory glass with the amount of the appropriate organic solvent, and the determination of non-encapsulated oil was continued. In case of FAMEs preparation for GC analysis, oil from the capsules was collected using a syringe with a needle from an appropriate amount of randomly selected hydrogel capsules.

#### 2.6.2. Determination of the Impact of Encapsulation Process on Oil Quality

The quality of encapsulated oil was tested directly after encapsulation to confirm the lack of a negative impact of this process on the oil. The non-encapsulated oils were used as control samples.

#### 2.6.3. Determination the Protective Role of Encapsulation on the Oil Quality during Storage

The assessment of the protective role of the capsule shell on the oxidative stability of the encapsulated oil was carried out using accelerated aging tests. Oil in the form of capsules was enclosed in glass bottles containing distilled water, which was necessary to maintain the structure of the hydrogel. Non-encapsulated oil, which was the reference sample, was closed in identical glass bottles in the amount corresponding to the oil closed in the capsules. The samples of non-capsulated oil and encapsulated oil were placed in a thermostatic chamber (Memmert HCP 108 GmbH + Co. GK, Schwabach, Germany) at 40 °C and stored for four weeks. The quality of the oil was tested after two and four weeks of storage, according to Section 2.6.1.

### 2.7. Water-Binding Capacity

The water-binding capacity (WBC) of chitosan and sodium alginate was measured using the modified method of Wang and Kinsella [26]. Water absorption was initially carried out by weighing a centrifuge tube containing 0.5 g of sample, adding 10 mL of water, and mixing it on a vortex mixer for one minute to disperse the sample. The contents were left at ambient temperature for 30 minutes, with shaking for five seconds every 10 minutes, and were then centrifuged at 3200 rpm for 25 minutes. After the supernatant was decanted, the tube was weighed again. The WBC was calculated as follows:

WBC (%) [water bound (g)/sample weight (g)] × 100(3)

### 2.8. Biocompatibility Tests

#### 2.8.1. Cell Culture

L929 cells (adult mouse fibroblast cell line) were obtained from ATCC (American Type Culture Collection). Cells (mycoplasma free) were maintained in a monolayer culture at 37 °C in a humidified 5% CO_2_ atmosphere in low-glucose Dulbecco’s modified Eagles medium (DMEM) supplemented with 10% fetal bovine serum (FBS), 100 units/mL of penicillin, 100 µg/mL of streptomycin, and 10 mM of glutamine (Sigma-Aldrich, St. Louise, CA, USA). Under these conditions, the cell-doubling time was 24 h.

#### 2.8.2. Cell Viability Assessment

The cytotoxicity of the composite chitozan was assessed by 3-(4,5-dimethylthiazol-2-yl)-2,5-diphenyltetrazolium bromide (MTT) assay according to the ISO 10993-5:2009(E) [27]. L929 cells at a density of 1 × 10^4^ cells/well were seeded on a 96-well tissue culture microtiter plate and a 100-µL culture medium only (blank) was dispensed into peripheral wells. The cells were incubated for 24 h (5% CO_2_, 37 °C, >90% humidity) to form a half-confluent monolayer. After 24 h of incubation, the culture medium was aspirated from the cells, and 100 µL of treatment medium containing either the appropriate concentration of samples, the positive control, or blank was added. After 24 h of incubation, the cell viability was examined. The attached and proliferated cells were quantified using MTT assay. Briefly, the culture medium was removed, and 50 µL of MTT solution (one mg/mL in medium without supplements and phenol red) was added to each well and incubated for two hours at 37 °C in a humidified 5% CO_2_ atmosphere. After that, the media with MTT solution was removed, and crystals of formazan from each well were suspended in 100 µL of isopropanol. The plate was left on a shaking platform for 10 minutes. The absorbance was recorded on a Microplate Reader (Bio-Rad, Hercules, CA, USA) at the length of 570 nm. The experiments were repeated at least three times. A decrease in the number of living cells correlated to the amount of blue-violet formazan formed, as monitored by the optical density at 570 nm. To calculate the reduction of viability compared to the blank, the following equation was used:

Viability% = 100 × *OD*_570*e*_/*OD*_570*pc*_ (4)

where: *OD*_570*e*_ is the mean value of the optical density of the samples; and *OD*_570*pc*_ is the mean value of the optical density of the positive control (L929 cells in culture medium).

### 2.9. Statistical Analysis of the Data

The presented results are the average values from at least three replications. The data were evaluated by analysis of variance (one-way ANOVA procedure) using the program SigmaPlot 11.0 (Systat Software, Erkrath, Germany), and the differences between the means were determined by Tukey’s multiple test (*p* < 0.05).

## 3. Results

The formation of the capsules may be affected by many variables resulting from equipment construction and the physicochemical properties of encapsulated substances and media for their immobilization, such as: viscosity, surface tension of the core and shell solution, and the interactions between them.

The polymer capsules were created using the drop-in-drop coaxial method. For the gelation of the chitosan and sodium alginate capsules, alcoholic solutions of sodium hydroxide and calcium lactate were used, respectively. Sodium hydroxide causes the fall out of the chitosan from the solution as a microcrystalline precipitate, while the presence of ethyl alcohol significantly accelerates this process [28]. This composition of the solution allows avoiding oil leakage from the capsule after falling into the hardening solution. The fast sodium alginate gelation reaction occurs in the presence of calcium ions, creating a molecular structure called the egg-box model [29]. An additional advantage of the lactate form of the calcium salt is the lack of influence on taste.

The particle size distribution measurement showed that the chitosan capsules’ diameter was in the range of 3.9 ÷ 4.3 mm, where the capsules with diameters of 3.9, 4.0, 4.1, 4.2, and 4.3 mm represented 1.4 ± 0.2%, 3.4 ± 0.4%, 8.0 ± 1.0%, 83.7 ± 3.4%, and 4.1 ± 0.2% of the studied population, respectively. This range is consistent with the results that we obtained in our earlier work [10]. In the case of capsules with a sodium alginate shell, the diameter range was 3.7 ÷ 4.1 mm. The alginate capsules with diameters of 3.7, 3.8, 3.9, 4.0, and 4.1 mm represented 0.9 ± 0.1%, 4.5 ± 0.1%, 15.7 ± 1.6%, 78.7 ± 4.0%, and 1.8 ± 0.2% of the studied population, respectively.

Table 1 shows that the encapsulation efficiency was at least 90%, and did not differ significantly, depending on the composition of the core or capsule shell.

Relative to the review work of Bakry et al. [8], this is a value higher than that obtained during encapsulation by emulsification and interfacial polymerization, and also comparable with the encapsulation efficiency using extrusion, spray drying, or fluidized bed-coating methods. The hydrogel capsules contained 38–45% oil, where the chitosan capsules were characterized by a higher oil content in the core of about 10% compared to alginate capsules. This is the small, but significant difference that can be related to the viscosity and surface tension of the shell polymer solutions [30]. A 2% chitosan solution has a higher viscosity than a 2% solution of sodium alginate, which may indicate that the first one is less susceptible to shear forces associated with dispensing oil into the shell solution droplets, and is more efficient at filling with the same encapsulation efficiency.

### 3.1. Impact of Encapsulation Process on the Oil Quality

The research material was the refined rapeseed oil “Kujawski”, having at least one year’s shelf-life, and the cold-pressed, high-linoleic linseed oil “Wielkopolski”, for which the shelf-life date was at least half a year. In all of the tests, homogeneous batches of rapeseed oil and linseed oil were obtained by combining and mixing five liters of oil from smaller unit packages. The prepared oil samples were encapsulated using the coaxial method according to Section 2.3 [10].

**Figure 1:**
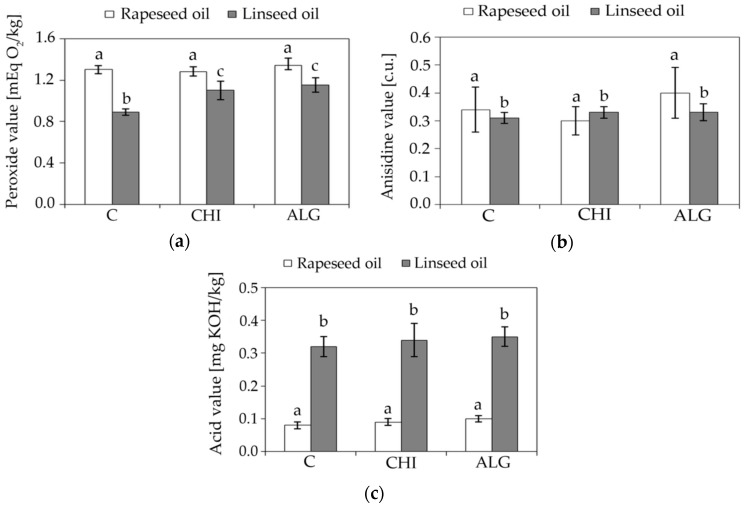
Comparison of the content of the: (**a)** primary and (**b**) secondary oxidation products and (**c**) the content of free fatty acids in non-encapsulated oils and in oils immediately after encapsulation, where C—non-encapsulated oil, CHI—oil encapsulated in chitosan shell, and ALG—oil encapsulated in alginate shell. Values marked with the same letters do not differ significantly (n = 3, *p* < 0.05).

The quality of each oil (rapeseed and linseed, encapsulated and before encapsulation) has been examined based on the peroxide, anisidine, and acid values. Figure 1 shows that the encapsulation did not significantly affect the content of the primary oxidation products in rapeseed oil. In linseed oil, the peroxide value increased by approximately 20% relative to the oil sample prior to encapsulation. Nevertheless, the peroxide values of linseed oil before and after the process were lower compared to the corresponding samples of rapeseed oil (Figure 1a).

In addition to the undesirable substances, many substances with antioxidant properties (e.g., tocopherols, carotenoids, sterols) were removed from the oil during the refining process, which could be the reason for this situation [31]. In all of the tests, the peroxide value did not exceed the maximum allowable limit of five mEq O_2_/kg for refined rapeseed oil [32] and linseed oil as cold-pressed fat equal to 15 mEq O_2_/kg oil [33]. All of the tested oil samples were characterized by a low and similar anisidine value (Figure 1b). Makareviciene and Janulis [34] stated that the level of this value for refined oils should not exceed eight units. According to the same authors, the maximum AV for virgin oils should not exceed three units, while Subramanian et al. [35] claimed that the upper limit of the *p*-anisidinenumber has not been determined so far exclusively for cold-pressed oils, and should be not higher than two units. In none of the tested samples was the limit exceeded (Figure 1b). It can be concluded that the encapsulation did not result in a statistically significant change in the content of secondary products of the two tested oils, irrespective of the type of polymer used to create the capsule shell. The encapsulation also did not change the content of free fatty acids in the analyzed oils. Both before and after encapsulation, these values were the same for oils of the same type (Figure 1c).

The permissible value of the free fatty acid has not been exceeded for either rapeseed oil samples: 0.3 mg KOH/kg of oil [32] and for linseed oil samples: 4.0 mg KOH/kg of fat, for which the quality requirements are identical with the quality requirements for cold-pressed vegetable oils from various raw materials [33]. Higher values of the acid number of linseed oil than rapeseed oil can be explained by the rapeseed oil refining step, during which free fatty acids are effectively removed from the oil [36].

### 3.2. Protective Role of Encapsulation on the Oil Quality during Storage

The rapeseed oil and linseed oil that were prepared for determination in accordance with the procedure described in Section 3.1 were used to carry out the experiment. The prepared oil samples were encapsulated using the coaxial method according to Section 2.3. The quality of oil (rapeseed and linseed, encapsulated and non-encapsulated) after two and four weeks storage has been examined based on the peroxide, anisidine, and acid values, as well as the fatty acid profile.

The PV of rapeseed oil and linseed oil in the control samples and encapsulated samples before the start of the test was small (Table 2) and comparable to the values measured immediately after encapsulation in the previous test (Figure 1a).

The highest PV of linseed oil was obtained after four weeks of storage for the non-encapsulated sample. Moreover, it was the highest value of all of the samples determined after this time. For the linseed oil sample stored under the same conditions in the form of alginate capsules, the result was 20% lower. The greatest protective effect in the test was obtained by encapsulating the linseed oil in chitosan hydrogel. In this case, the PV was 88% lower than the value obtained for the control sample (Table 2). It was the only sample in which the permissible value of the peroxide number determining the possibility of oil consumption was not exceeded. The highest PV of rapeseed oil was obtained in the control sample after four weeks of storage. The content of peroxides in the rapeseed oil that was stored for four weeks in the form of chitosan and alginate capsules was 40% lower than that in the control sample (Table 2).

The analyzed samples showed different levels of secondary oxidation products (SOPs) depending on the type of oil and its storage form. In each of the samples, the anisidine value increased during the storage time. The highest AV after the end of the test was obtained for control samples, rapeseed oil, and linseed oil (Table 3).

In turn, the lowest value was obtained for linseed oil stored in the form of chitosan capsules, and rapeseed oil stored in the form of alginate capsules (Table 3). In the case of the linseed oil encapsulated in sodium alginate, the SOPs content was approximately 20% higher than in the oil inside the chitosan shell. In the case of rapeseed oil, encapsulation in the chitosan polymer caused the generation of only 5% more SOPs than in the alginate one.

The content of free fatty acids (FFAs) in rapeseed oil was low in the control and encapsulated samples. After four weeks of storage at 40 °C, the FFAs’ content increased equally, regardless of the oil form that was used in the test (Table 4). This means that the encapsulation stage does not contribute to the formation of free fatty acids. What’s more, the AcV values after the test fulfilled the requirements [32], according to which the permissible value for refined rapeseed oil does not exceed 0.3 mg KOH/kg. The quality requirements for cold-pressed linseed oil are identical to the quality requirements for cold-pressed vegetable oils from various raw materials: 4.0 mg KOH/kg [33]. The content of FFAs in non-encapsulated and encapsulated linseed oil was similar during the test and after it was finished (Table 4). The free fatty acids are removed during refining oil at the deacidification stage in the form of soaps; hence, their higher content in linseed oil is fully justified [36].

In both oils, the total content of saturated fatty acids (SFAs) was in the range of 8–10%. Both oils were also characterized by a high and similar share of unsaturated acids, with 60–65% content of monounsaturated fatty acids (MUFAs) and 25–30% content of polyunsaturated acids (PUFAs) in rapeseed oil. In the case of linseed oil, the content of MUFAs and PUFAs was exactly the opposite of the amounts that were measured for the rapeseed oil (Table 5 and Table 6).

The dominant fatty acids in rapeseed and linseed oil were oleic acid and linolenic acid, respectively. The content of other acids was typical for the raw materials from which they were obtained, and consistent with the data available in the scientific literature [37,38]. Table 5 and Table 6 present the changes in the composition of fatty acids that occurred in rapeseed and linseed oil during storage in unencapsulated form and in the form of chitosan and alginate capsules.

The results indicate that statistically significant changes in the fatty acids profile in rapeseed oil occurred only in the case of the non-encapsulated form of oil. These changes consisted in an increase of the SFAs content (10%), which was mainly palmitic and stearic acid, and the decrease in the content of MUFAs and PUFAs (each 5%). In the samples that were stored for four weeks at 40 °C in encapsulated form, no significant changes were observed in the fatty acids profile. The content of trans fatty acids listed in the table also did not change (Table 5).

A similar relationship regarding the change of the fatty acids profile was observed in the case of linseed oil. The statistically significant changes concerned an increase in the content of SFAs (7.5%) and a decrease in the content of MUFAs (3.5%) and PUFAs (1.3%). In samples stored for four weeks at 40 °C in encapsulated form, no significant changes were observed in the fatty acids profile. The content of trans fatty acids listed in the table had also not changed (Table 6). Fatty acids composition is one of the most important factors that determine the oxidative stability of the oils. It is known that the rate of C18:2 acid oxidation is 10–40 times higher than that of C18:1, and the rate of C18:3 oxidation is two to four times faster than that of C18:2 [39]. According to this relationship, changes in the composition of fatty acids should be more noticeable in the case of linseed oil.

In a study of White et al. [40], the composition of linseed oil changed only slightly during storage over six months. Only the increase in the amount of palmitic acid was statistically significant, e.g., from 6.6% to 7.0% in variety Linola^TM^ 947. The above results are in confirmation with the findings of Ayton et al. [41], who reported a slight increase of SFAs and decrease of PUFAs up to 1.3%, in an olive oil sample. The content of individual fatty acids did not change significantly only in the samples encapsulated regarding the control oils, which partly confirms to a certain extent the protective function of the hydrogel membrane made of the chitosan or sodium alginate. However, the measurement of changes in the composition of fatty acids can not be in this case a clear indicator of the capsules’ protective role. The data that are available in the scientific literature regarding changes in the fatty acids profile during the storage of edible oils are not entirely predictable. They are the resultant of factors, such as fatty acids composition, time and temperature of storage, the presence of different compounds in oil affecting the rate of radical generation, or the type of food in which the oil was present. For example, changes that occurred in the composition of fatty acids in frozen calves’ meat stored for three months, due to the increase of PUFAs level, are inconsistent with the mechanism of fat autooxidation [42].

Both oils before encapsulation were characterized by a similar content of tocopherols, which was expressed as the sum of these compounds (Table 7).

The content of tocopherols in rapeseed and linseed oil before the start of the test is comparable with the data contained in other works [43,44,45]. After the test, non-encapsulated rapeseed oil was characterized by a 44% lower content of total tocopherols, of which 37% was the reduction of the form alpha. In the rapeseed oil encapsulated in the chitosan and alginate membranes, the content of tocopherols decreased by 5% and 16%, respectively (Table 7). In the case of linseed oil, the reduction of total tocopherol content for the control, and CHI and ALG samples, was 21%, 7%, and 11%, respectively. It was found that the singlet oxygen oxidation rate of tocopherols varied in the order α > γ > δ [46], which is also confirmed by our results. Goffman and Möllers [47], in research on the stability of tocopherols in rapeseed oil, showed that individual tocopherols do not degrade in 5 °C and 20 °C during 24 weeks of storage. However, at 40 °C, degradation was already observed after four to six weeks.

Wagner and Elmadfa [48] showed the stabilizing effect of tocopherols and their mixtures (100 mg/100 g oil) in linseed oil: γ-T > γ/δ-T > δ-T > γ/α-T > α-T> α/δ-T. So, the greater protective effect of encapsulation observed in the case of linseed oil may be the result of a synergistic action of two factors. The first is the protective role of polymers in the form of hydrogel shells acting as a physical barrier limiting oxygen access to the inside of capsules, where the solubility of oxygen in membrane is similar to water, and about 10 times lower than in edible oil (3 mol/L in oil) [49]. The second is the higher content of a more stable γ-tocopherol form in linseed oil (over 90% of total tocopherol value).

Tańska et al. [50] showed a high positive correlation between peroxide value and dienes content (>0.82) in rapeseed and linseed oil, which means that the largest increase in dienes should be in oil samples without encapsulation. The obtained results confirm the above correlation (Table 8).

The content of conjugated dienes and trienes in the samples of linseed and rapeseed oil before storage and after the test in the form of capsules did not statistically differ, and was comparable to result obtained in similar works (Table 5) [31,50,51]. From the statistical point of view, the obtained results may indicate the slower unfavorable changes of encapsulated oil, especially linseed oil with a high content of linolenic acid.

### 3.3. Biocompatibility Test

**Figure 2:**
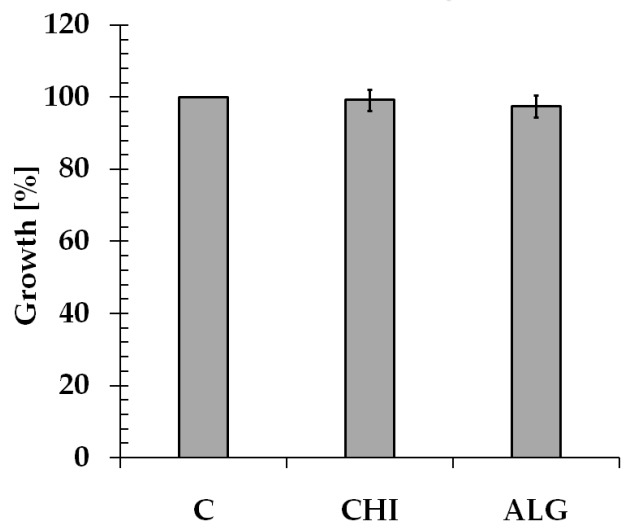
The 3-(4,5-dimethylthiazol-2-yl)-2,5-diphenyltetrazolium bromide (MTT) viability assay of chitosan (CHI) and sodium alginate (ALG) samples following 24 h of incubation with L929 cells. The mean ± SD values from three independent experiments (n = 3, *p* < 0.05).

The in vitro cytotoxicity of chitosan and sodium alginate materials was investigated by the MTT assay using adult mouse fibroblast L929 cells according to ISO 10993-5:2009(E) [27]. This is a standard in vitro method for the biological evaluation of medical devices, which relies on the mitochondrial activity of vital cells, and represents a parameter for their metabolic activity [52]. The results are shown in Figure 2, and are expressed as a percentage of viable cells in the presence of the test of the chitosan and sodium alginate versus control without the material. It was shown that the tested samples did not cause the inhibition of cell growth compared to the control.

## 4. Discussion

Stability tests confirmed that the encapsulation step of rapeseed and linseed oil using the coaxial technique does not cause the deterioration of the quality of oils evaluated immediately after encapsulation, regardless of the biopolymer used in the function of the capsule shell. The biggest protective role showed the chitosan in a system with linseed oil. Moreover, the PV, AV, and AcV of linseed oil encapsulated in chitosan membrane after the test met the requirements determining the suitability of the oil for consumption.

The outperforming effect of chitosan over alginate may be the result of the different oxygen permeability of used biopolymers. The available scientific papers confirm that the oxygen is able to penetrate through chitosan and sodium alginate films [53,54], but it was also noted that films made from proteins and carbohydrates are excellent barriers to oxygen, because of their tightly packed, ordered hydrogen-bonded network structure. However, information about the lower oxygen solubility of chitosan compared with alginate membranes is also confirmed [55]. Caner et al., in studies on chitosan films, showed that oxygen permeability does not depend on the storage time of the materials, but rather depends on the presence and type of acid used to dissolve the chitosan and its concentration [56]. Oxygen permeability is dependent on the relative humidity [57]. As the relative humidity increases, more water molecules interact with the material, and the film becomes more plasticized. In these conditions, the mobility and the extensive mass transfer across the film are favored. 

Alginates are made up of 13-D mannuronic acid and ct-l-guluronic acid. These two units do not display a random distribution, but rather occur in blocks containing about 20 units, which associate at various degrees. The carboxyl groups that are present on each sugar unit of the chain contribute to bind water and promote strong electrostatic repulsion between the chains, which accounts for the high hydration of polymer, which is expressed as the water-binding capacity (WBC). The WBC of chitosan and sodium alginate that was determined by us was 10.23 ± 0.04 and 27.63 ± 0.06, respectively.

The WBC parameter of sodium alginate, as reported by Sánchez, was equal to 25 ± 2.4 mL/g [58]. According to the research of Cho et al., the WBC value of chitosans with different deacetylation degrees was in the range of 4.6–8.1 mL/g [59]. This may be the reason for limiting the oxygen route through the polymer membrane.

Castejón et al. demonstrated the relationship between the microstructure and the air permeability of polymeric membranes, where the air permeability decreases with the decreasing porosity of the membranes [60]. This is consistent with the water-binding theory and publications confirming the usually smaller porosity of chitosan membranes compared to alginate ones [61].

The obtained results indicate that each encapsulation system should be optimized in view of the properties of the encapsulated compounds especially regarding the oils susceptible to xidation processes, which are reactions difficult to control. The coaxial encapsulation system in the form of a battery can be a powerful tool for obtaining new forms of hydrogel capsules that prolong the freshness of oils. The use of the physicochemical properties of chitosan and alginate allows creating an effective hydrogel protective membrane limiting oxidation processes, which can be used not only to encapsulate oils, but also for many other dietary supplements with lipophilic properties.

## Figures and Tables

**Table 1:** Comparison of the encapsulation efficiency, the content of oil in capsules, and the oil-to-polymer mass ratio, depending on the shell and the core composition of the capsules.

Capsule Shell	Chitosan		Sodium Alginate	
Capsule Core	Rapeseed Oil	Linseed Oil	Rapeseed Oil	Linseed Oil
Ecapsulation efficiency [%]	94.2 ± 2.5 ^a^	93.2 ± 1.6 ^a^	91.6 ± 0.9 ^a^	91.0 ± 1.1 ^a^
Oil content [%]	44.6 ± 0.8 ^a^	43.5 ± 0.9 ^a^	39.9 ± 1.0 ^b^	38.4 ± 0.8 ^b^
Oil to polymer ratio [g/g]	34.8 ± 0.3 ^a^	34.0 ± 0.5 ^a^	31.2 ± 0.5 ^b^	29.8 ± 0.4 ^b^

Values in individual lines marked with the same letters do not differ significantly (*p* < 0.05, n = 3).

**Table 2:** Comparison of the change of the content of primary oxidation products in non-encapsulated and encapsulated rapeseed and linseed oil during four weeks of storage at 40 °C (C—non-encapsulated oil, CHI—oil encapsulated with chitosan, ALG—oil encapsulated with sodium alginate).

Sample	Oil	Time of Storage at 40 °C [weeks]		
		0	2	4
C	Linseed	0.89 ± 0.03 ^a^	10.64 ± 0.24 ^a^	65.07 ± 1.53 ^a^
CHI		1.10 ± 0.09 ^b^	3.94 ± 0.15 ^b^	7.48 ± 0.35 ^b^
ALG		1.15 ± 0.07 ^b^	8.47 ± 0.20 ^c^	48.25 ± 1.48 ^c^
C	Rapeseed	1.30 ± 0.04 ^a^	11.21± 0.17 ^a^	35.52 ± 1.98 ^a^
CHI		1.28 ± 0.05 ^a^	8.74 ± 0.78 ^b^	19.92 ± 1.69 ^b^
ALG		1.34 ± 0.07 ^a^	10.55 ± 0.81 ^c^	20.77 ± 1.02 ^c^

Values in individual lines marked with the same letters do not differ significantly (*p* < 0.05, n = 3).

**Table 3:** Comparison of the change of the content of secondary oxidation products (SOPs) in non-encapsulated and encapsulated rapeseed and linseed oil during four weeks of storage at 40 °C (C—non-encapsulated oil, CHI—oil encapsulated with chitosan, ALG—oil encapsulated with sodium alginate).

Sample	Oil	Time of Storage at 40 °C [weeks]		
		0	2	4
C	Linseed	0.31 ± 0.02 ^a^	2.72 ± 0.21 ^a^	9.34 ± 0.19 ^a^
CHI		0.33 ± 0.02 ^a^	1.15 ± 0.12 ^b^	3.02 ± 0.31 ^b^
ALG		0.33 ± 0.03 ^a^	1.20 ± 0.09 ^b^	5.05 ± 0.34 ^c^
C	Rapeseed	0.34 ± 0.08 ^a^	4.56 ± 0.42 ^a^	11.30 ± 0.90 ^a^
CHI		0.30 ± 0.05 ^a^	3.41± 0.22 ^b^	7.52 ± 1.05 ^b^
ALG		0.40 ± 0.09 ^a^	3.27 ± 0.25 ^b^	6.98 ± 0.87 ^b^

Values in individual lines marked with the same letters do not differ significantly (*p* < 0.05, n = 3).

**Table 4:** Comparison of the change of the free fatty acids in non-encapsulated and encapsulated rapeseed and linseed oil during four weeks of storage at 40 °C (C—non-encapsulated oil, CHI—oil encapsulated with chitosan, ALG—oil encapsulated with sodium alginate).

Sample	Oil	Time of Storage at 40 °C [weeks]		
		0	2	4
C	Linseed	0.32 ± 0.03 ^a^	0.87 ± 0.01 ^a^	1.22 ± 0.10 ^a^
CHI		0.34 ± 0.05 ^a^	0.92 ± 0.05 ^a^	1.34 ± 0.09 ^a^
ALG		0.35 ± 0.03 ^a^	1.10 ± 0.04 ^b^	1.25 ± 0.11 ^a^
C	Rapeseed	0.08 ± 0.02 ^a^	0.15 ± 0.03 ^a^	0.30 ± 0.04 ^a^
CHI		0.09 ± 0.02 ^a^	0.16 ± 0.03 ^a^	0.29 ± 0.02 ^a^
ALG		0.10 ± 0.03 ^a^	0.13 ± 0.04 ^a^	0.32 ± 0.03 ^a^

Values in individual lines marked with the same letters do not differ significantly (*p* < 0.05, n = 3).

**Table 5:** Comparison of the composition of the most important fatty acids of rapeseed oil during four weeks of storage in non-encapsulated form and in the form of chitosan and alginate capsules.

Type of Fatty Acid	Mean Value [% m/m]			
	Oil before Test	Oil after Four Weeks of Storage at 40 °C		
		C	CHI	ALG
C16:0	4.82 ± 0.04 ^a^	5.33 ± 0.08 ^b^	4.89 ± 0.03 ^a^	5.01± 0.15 ^a^
C18:0	1.60 ± 0.04 ^a^	1.88 ± 0.03 ^b^	1.73 ± 0.09 ^a^	1.68 ± 0.07 ^a^
C18:1 (*trans*)	0.03 ± 0.01 ^a^	0.02 ± 0.00 ^a^	0.03 ± 0.01 ^a^	0.02 ± 0.01 ^a^
C18:1 (*cis*)	59.8 ± 0.74 ^a^	57.4 ± 0.38 ^b^	59.0 ± 0.82 ^a^	58.5 ± 0.61 ^a^
C18:2 (*trans*)	0.05 ± 0.01 ^a^	0.03 ± 0.01 ^a^	0.02 ± 0.00 ^a^	0.06 ± 0.01 ^a^
C18:2 (cis)	18.79 ± 0.37 ^a^	17.0 ± 0.25 ^b^	18.42 ± 0.14 ^a^	18.36 ± 0.20 ^a^
C18:3	9.64 ± 0.13 ^a^	10.0 ± 0.27 ^a^	9.71 ± 0.16 ^a^	9.60 ± 0.29 ^a^
C20:0	0.32 ± 0.08 ^a^	0.23 ± 0.04 ^a^	0.15 ± 0.09 ^a^	0.18 ± 0.04 ^a^
C20:1	2.70 ± 0.11 ^a^	2.50 ± 0.15 ^a^	2.71± 0.07 ^a^	2.65 ± 0.08 ^a^
C22:0	0.83 ± 0.18 ^a^	1.19 ± 0.26 ^a^	0.95 ± 0.14 ^a^	0.83 ± 0.12 ^a^
C22:1	1.54 ± 0.09 ^a^	0.93 ± 0.07 ^b^	1.32 ± 0.17 ^a^	1.46 ± 0.10 ^a^
ΣSFA	7.57± 0.17 ^a^	8.63 ± 0.11 ^b^	7.72 ± 0.10 ^a^	7.70 ± 0.13 ^a^
ΣMUFA	64.07 ± 1.02 ^a^	60,85 ± 0.87 ^b^	63.06 ± 0.95 ^a^	62,63 ± 1.11 ^a^
ΣPUFA	28.48 ± 0.20 ^a^	27.03 ± 0.09 ^b^	28.15 ± 0.13 ^a^	28.02 ± 0.09 ^a^

Values in individual lines marked with the same letters do not differ significantly (*p* < 0.05, n = 3).

**Table 6:** Comparison of the composition of the most important fatty acids of linseed oil during four weeks of storage in non-encapsulated form and in the form of chitosan and alginate capsules.

Type of Fatty Acid	Mean Value [% m/m]			
	Oil before Test	Oil after Four Weeks of Storage at 40 °C		
		C	CHI	ALG
C16:0	4.80 ± 0.09 ^a^	5.08± 0.11 ^b^	4.89 ± 0.05 ^a^	4.90 ± 0.08 ^a^
C18:0	5.01 ± 0.03 ^a^	5.51 ± 0.05 ^b^	5.10 ± 0.03 ^a^	5.08 ± 0.06 ^a^
C18:1 (*trans*)	Nd ^a^	Nd ^a^	Nd ^a^	Nd ^a^
C18:1 (*cis*)	24.52 ± 0.56 ^a^	25.51 ± 0.62 ^a^	25.05 ± 0.33 ^a^	24.57 ± 0.47 ^a^
C18:2 (*trans*)	0.05 ± 0.01 ^a^	0.02 ± 0.00 ^a^	0.03 ± 0.01 ^a^	0.05 ± 0.01 ^a^
C18:2 (cis)	18.13 ± 0.23 ^a^	19.93 ± 0. 15 ^b^	18.66 ± 0.32 ^a^	18.71 ± 0.58 ^a^
C18:3	45.79 ± 0.83 ^a^	43.19 ± 0.41 ^b^	45.00 ± 0.50 ^a^	44.61 ± 0.37 ^a^
C20:0	0.07 ± 0.01 ^a^	0.10 ± 0.02 ^a^	0.05 ± 0.01 ^a^	0.06± 0.01 ^a^
C20:1	0.08 ± 0.02 ^a^	Nd ^b^	0.05 ± 0.01 ^a^	0.06 ± 0.01 ^a^
C22:0	Nd ^a^	Nd ^a^	Nd ^a^	Nd ^a^
C22:1	Nd ^a^	Nd ^a^	Nd ^a^	Nd ^a^
ΣSFA	9.88 ± 0.13 ^a^	10.69 ± 0.09 ^b^	10.04 ± 0.08 ^a^	10.04 ± 0.05 ^a^
ΣMUFA	24.60 ± 0.28 ^a^	25.51± 0.31 ^b^	25.10 ± 0.12 ^b^	24.63 ± 0.11 ^a^
ΣPUFA	63.97 ± 1.21 ^a^	63,14 ± 1.14 ^a^	63.69 ± 0.99 ^a^	63.37 ± 0.93 ^a^

Nd—not detected. Values in individual lines marked with the same letters do not differ significantly (*p* < 0.05, n = 3).

**Table 7:** Comparison of tocopherols content in rapeseed and linseed oils.

Sample			Content of Tocopherol (mg/100 g)				
			α	ß	γ	δ	Total *
Rapeseed oil	Before test		15.3 ± 3.2 ^a^	Nd ^a^	28.1 ± 2.3 ^a^	1.4 ± 0.2 ^a^	44.8
	After four weeks of storage at 40 °C	C	2.4 ± 2.0 ^b^	Nd ^a^	22.5 ± 1.7 ^b^	Nd ^b^	24.9
		CHI	12.0 ± 1.5 ^c^	Nd ^a^	29.5 ± 1.9 ^a^	1.0 ± 0.3 ^a^	42.5
		ALG	10.7 ± 3.2 ^c^	Nd ^a^	25.9 ± 2.6 ^a^	1.2 ± 0.2 ^a^	37,8
Linseed oil	Before test		0.5 ± 0.1 ^a^	Nd ^a^	42.3 ± 2.8 ^a^	1.0 ± 0.2 ^a^	43.8
	After four weeks of storage at 40 °C	C	Nd ^b^	Nd ^a^	34.5 ± 1.9 ^b^	Nd ^a^	34.5
		CHI	Nd ^b^	Nd ^a^	40.6 ± 3.2 ^a^	1.2 ^a^	41.8
		ALG	Nd ^b^	Nd ^a^	39.1 ± 1.4 ^a^	Nd ^a^	39.1

Values in individual columns for each oil marked with the same letters do not differ significantly (*p* < 0.05, n = 3). Nd—not detected, * value calculated mathematically by summing the values of all forms of tocopherols.

**Table 8:** Comparison of dienes and trienes content in rapeseed and linseed oils.

Sample			Dienes (%)	Trienes (%)
Rapeseed oil	Before test		0.12 ± 0.03 ^a^	0.02 ± 0.01 ^a^
	After four weeks of storage at 40 °C	C	0.23 ± 0.02 ^b^	0.05 ± 0.02 ^a^
		CHI	0.10 ± 0.03 ^a^	0.03 ± 0.01 ^a^
		ALG	0.13 ± 0.02 ^a^	0.02 ± 0.01 ^a^
Linseed oil	Before test		1.90 ± 0.02 ^a^	0.15 ± 0.4 ^a^
	After four weeks of storage at 40 °C	C	2.43 ± 0.04 ^b^	0.32 ± 0.3 ^b^
		CHI	1.99 ± 0.07 ^a^	0.14 ± 0.5 ^a^
		ALG	2.05 ± 0.06 ^a^	0.19 ± 0.3 ^a^

Values in individual columns for each oil marked with the same letters do not differ significantly (*p* < 0.05, n = 3).

## Supplementary Materials

The following are available online at http://www.mdpi.com/2073-4360/10/12/1355/s1, Video S1: Presentation the principle of drop-in-drop encapsulation method using chitosan or alginate solution.

## List of Symbols

AcV	Acid value
AV	Anisidine value
DMEM	Low Glucose Dulbecco’s modified Eagles’ medium
FBS	Fetal Bovine Serum
FFA	Free fatty acid
MTT	3-(4,5-dimethylthiazol-2-yl)-2,5-diphenyltetrazolium bromide
MUFA	Monounsaturated fatty acid
PUFA	Polyunsaturated fatty acid
PV	Peroxide value
SFA	Saturated fatty acid
SOPs	Secondary oxidation products
WBC	Water binding capacity
α-T	α-tocopherol
γ-T	γ-tocopherol
δ-T	δ-tocopherol
